# Effect of sodium butyrate regulating IRAK1 (interleukin-1 receptor-associated kinase 1) on visceral hypersensitivity in irritable bowel syndrome and its mechanism

**DOI:** 10.1080/21655979.2021.1920324

**Published:** 2021-04-27

**Authors:** Yuqin He, Yan Tan, Jianru Zhu, Xiaofeng Wu, Zhiyong Huang, Hengsheng Chen, Min Yang, Dongfeng Chen

**Affiliations:** a Department of Gastroenterology, Daping Hospital, Army Medical University, Chongqing, PR China; b Department of Pathophysiology, High Altitude Military Medicine, Daping Hospital, Army Medical University, Chongqing, PR China; c Department of Stem Cell and Regenerative Medicine, Gastroenterology, Daping Hospital, Army Medical University, Chongqing, PR China; d School of Microelectronics and Communication Engineering, Chongqing University, Chongqing, PR China; e Department of Neuroelectrophysiology, Children’s Hospital Affiliated to Chongqing Medical University, Chongqing, PR China

**Keywords:** Sodium butyrate, IRAK1, IBS, visceral hypersensitivity, mechanism

## Abstract

The current study aimed to investigate the effects of sodium butyrate on the level of colonic protein IRAK1 (interleukin-1 receptor-associated kinase 1) in irritable bowel syndrome (IBS) models as well as revealing the relationship between IRAKI level and visceral sensitivity during the progression of IBS. IBS symptoms were induced using TNBS (2,4,6-trinitrobenzene sulfonic acid) in mice and using IL-33 in HT-29 cells, which were then hanlded with sodium butyrate (100 mM for each mice and 0.05 M for HT-29 cells). The threshold of visceral pain and the expression of IRAKI in mice, and the level of IRAKI in HT-29 cells were detected. The data showed that the level of IRAK1 in IBS mice was higher than that in the control group, while the pre-treatment with sodium butyrate could solidy suppressed the level of IRAK1. Morevoer, it was found that the level of IRAK1 was negatively correlated with the pain threshold. In in vitro assays, the level of IRAK1 was firstly induced by IL-33 stimulation and then suppressed by sodium butyrate pretreatment. Collectively, the level of IRAKI showed an obvioulty positive relation with visceral hypersensitivity in IBS models, and the treatment with sodium butyrate could alleviate visceral hypersensitivity by inhibiting the expression of IRAKI.

## Graphical Abstract

**Figure:**
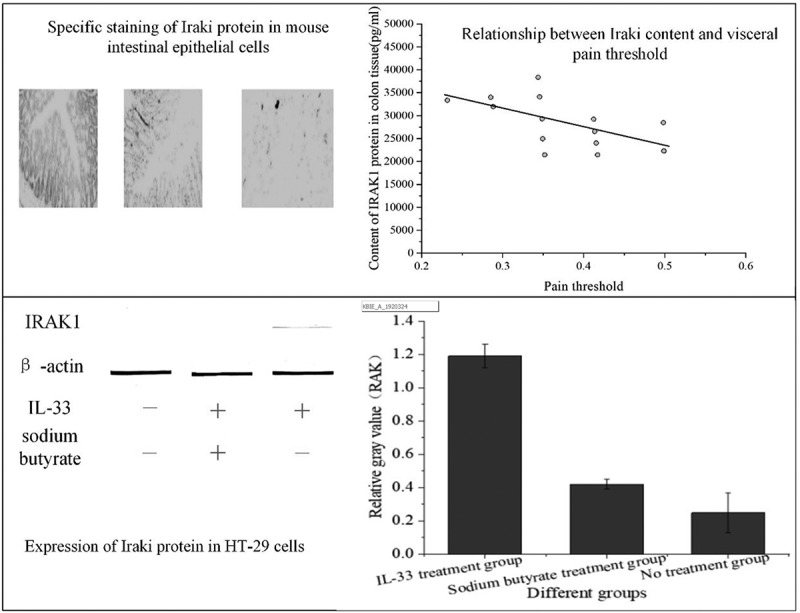

## 1. Introduction

Irritable bowel syndrome (IBS) is a relatively common functional gastrointestinal disease. The incidence of IBS in the world is about 3/20 of the total number in the world. Women and young people are prone to this disease [1,2]. The main symptoms of IBS are chronic and intermittent gastrointestinal dysfunction [3,4]. Kanazawa et al. (2017) [5] found that there were about 3/10–2/5 IBS patients with colorectal expansion with high sensitivity compared with normal people, mainly due to the decrease in visceral pain threshold and the improvement of the perception of dilation stimulation. However, the mechanism of visceral hypersensitivity in IBS patients is unclear. Ura (2016) et al. [6] found that some visceral hypersensitivity related to IBS may be associated with increased intestinal barrier permeability. The function of the intestinal barrier can ensure intestinal homeostasis and prevent pathogens and cytokines from entering the body. Some IBS’ intestinal micro inflammation may be an important factor to damage the function of the intestinal barrier and improve visceral hypersensitivity [7].

Butyric acid is an important metabolite of intestinal flora, which can provide energy to intestinal epithelial cells, regulate the metabolic process of intestinal epithelial cells, and improve the protection ability of the intestinal barrier [8]. Butyric acid can inhibit the activation of pro-inflammatory pathway in human colonic epithelial cells, and down-regulate the expression of some inflammatory factors, such as TNF-α and TLR2 [9], which indicates that sodium butyrate may inhibit the intestinal barrier micro inflammatory environment, thus improving the visceral hypersensitivity of IBS. IRAK1 (interleukin-1 receptor-associated kinase 1) is a key innate immune signal regulator in the IRAK family. It can be expressed in most organs of the human body. The composition and structure of IRAK1 protein are an N-terminal death domain, C-terminal C1 and C2 domains, and a central kinase domain containing an activation loop. IRAK1 is located in many inflammatory signaling pathways related to innate immunity. It is activated and phosphorylated by upstream signals. Moreover, it also calls TRAF6 and activates downstream MAPK signaling pathways. As a member of the IL-1 family, IL-33 can bind to its receptor ST2 and recruit IRAK1 to activate downstream signaling pathways. Nasehi et al. (2016) [10] found that the expression of IRAK1 in the intestinal tract of neonatal mice changes, which can help neonatal mice establish a temporary intestinal immune stable state.

In view of the above situation, it is assumed that sodium butyrate has a regulatory effect on irritable bowel syndrome, and the purpose is to analyze the effect of sodium butyrate on IRAK1 at the cellular level. The effect of sodium butyrate on IRAK1 protein expression and visceral sensitivity in the colon of irritable bowel syndrome is explored. The following research is made.

1. IBS mouse model is established and sodium butyrate enema is used to investigate whether sodium butyrate can regulate IRAK1.
2. The innovation of the exploration is to design cell experiments, select human colon cancer cell HT-29, and up regulate IRAK1 protein expression by IL-33 stimulation.
3. Whether the resistance of HT-29 cells to inflammatory factors changes after sodium butyrate pretreatment is analyzed to provide an experimental basis for exploring the mechanism of sodium butyrate in the treatment of IBS clinical symptoms.

## 2. Materials and methods

### 2.1. Subjects

Thirty healthy male mice (7 weeks, 20 g, purchased from the animal center of Xi’an Jiaotong University) were provided with diet and drinking water, and the feeding temperature was controlled at 21 °C. In 24 hours of a day, the day and night were replaced by 12 hours. The feeding process was under the national experimental animal feeding and use guidelines.

Grouping of experimental mice: Thirty mice were divided into IBS model group (10 mice, represented by letter A), sodium butyrate treatment group (10 mice, represented by letter B) and blank control group (10 mice, represented by letter C).

### 2.2. Preparation of mouse model

All the mice had fasted for 24 hours. The mice were anesthetized intraperitoneally with 1% Pentobarbital (Clontech company, USA) at the dose of 0.2 ml/mouse. The anal tube (after being coated with paraffin oil) was inserted into the anus of mice. When the distance between the end of the tube and the anal orifice was more than 6 cm, the anal tube was fixed on the tail of mice with adhesive tape. The mice in the IBS model group and sodium butyrate (Sigma company, USA) group were injected with 30% TNBS (2, 4, 6-trinitrobenzene sulfonic acid) solution. At a dose of 70 mg/kg, TNBS was slowly injected into the intestine of mice. During the injection, the anus of the mice should be kept elevated and the speed should be slow (to avoid the injection liquid overflowing from the anus). After 3 minutes, the anal tube was slowly pulled out and the mice were kept in an inverted position for 30 minutes. The blank control group was injected with the same dose of normal saline.

One week after TNBS injection, mice in the sodium butyrate group were enema with sodium butyrate (100 mol/L) at the dose of 0.3 ml/mouse, once every 2 days. IBS model group and blank control group were enemas with the same volume of normal saline. After 30 days of TNBS enema, the visceral hypersensitivity mice model of post-traumatic stress disorder (PTSD) was established by single prolonged stress (SPS) combined with restraint and plantar electric stimulation. Then, the intervention ended.

All mice were subjected to a colorectal distention test, and then the mice were killed by pentobarbital overdose anesthesia. The abdominal wall was quickly cut along the midline of the abdomen to expose the abdominal cavity. The distal colon was placed in the precooled normal saline, and the feces were washed off. Part of the distal colon was put into 4% paraformaldehyde (Beijing Solarbo Technology Co., Ltd., China). Two days later, at room temperature, the specimens were washed with running water for 5 hours, and then paraffin sections were prepared for immunohistochemical staining.

### 2.3. Colorectal distention test

The mice were fixed and their anus was fully exposed. The double lumen catheter coated with paraffin oil (Yangzhou Huayue Technology Development Co., Ltd., China) was slowly inserted into the mouse anus until the sacculus was about 2 cm away from the mouse anus. At the base of the mouse tail, the catheter was stabilized with adhesive tape. The mice were clamped with a large transparent beaker (perforated) to limit their activity area. A syringe (Shandong Zhushi Pharmaceutical Group Co., Ltd., China) was used to draw an appropriate amount of normal saline, and different volumes of normal saline (volume increased from 0 to 0.05 ml) were injected into the air sac (Shanghai yuanmu Biotechnology Co., Ltd., China). Each expansion time was maintained for 15 seconds (repeated for 3 times each expansion) until the mice developed pain reflex (abdominal wall tightening or tail tightening). The volume of normal saline at this time was recorded. The volume of normal saline that made the mice have pain reflex was the index of visceral sensitivity, that was, the threshold of visceral pain in mice.

### 2.4. Immunohistochemical staining

The prepared tissue slices were baked in an oven (Suzhou Zhongjie electric heating equipment Co., Ltd., China) at 65 °C for 1 h, and then dewaxing with xylene (Zhejiang Qianzun Medical Technology Co., Ltd., China). After alcohol dehydration, the slices were washed with distilled water and phosphate buffered saline (PBS) for 3 times, 5 minutes each time. The sections were placed in 0.01 M, pH6.0 sodium citrate buffer solution (Shanghai enzyme linked Biotechnology Co., Ltd., China), heated in the microwave oven at 92–98 °C for 10 minutes, and then cooled at room temperature. Then, the slices were rinsed with PBS for 3 times, 5 minutes each time. The slices were laid flat in the wet box. 5% goat serum blocking solution (Biyuntian Biotechnology Co., Ltd., China) was added to the slices and then the slices were sealed for half an hour at 37 °C. After goat serum was removed, anti IRAK1 antibody (human sample 1:500, mouse sample 1:1000) anti-p-IRAK1 antibody (1:200) (biyuntian Biotechnology Co., Ltd., China) was added overnight at 4 °C. After rewarming at room temperature for half an hour, PBS was used to rinse for 3 times, each time for 5 minutes. Mice and rabbit general second antibody (Tiangen Biochemical Technology Co., Ltd., China) were added, and the reaction time was 40 min at 37 °C. Then, PBS was used to rinse for 3 times, 5 min each time. Then, diaminobenzidine (DAB) ((alpha) Henan Weitixi Chemical Technology Co., Ltd., China) was used to color the tissue and then it was observed under a microscope (Olympus Corporation, Japan). After the appropriate time, the reaction was terminated. It was rinsed with tap water twice, 1 min each time. Hematoxylin (Beijing Solarbo Technology Co., Ltd., China) was used to re-dye the nucleus for 1 min, and then it was put into PBS for 8 min. Then, rapid dehydration and transparent treatment were carried out, and then neutral gum was used for sealing.

The samples were observed under a microscope. Five high-power fields were selected for each sample to evaluate the pathological changes in protein expression. The staining intensity was divided into 0–3 points (4 grades), followed by 0 points for negative, 1 point for weak positive, 2 points for moderate positive, and 3 points for strong positive cells. The percentage of positive staining cells was divided into 0–4 points (five grades). 0% was 0 point, 1%–25% was 1 point, 26%–50% was 2 points, 51%–75% was 3 points, and 76%–100% was 4 points. Expression grade = intensity score × positive cell count score. 0–3 points were negative expression, and 4–12 points were the positive expression.

### 2.5. Cell culture and intervention

After resuscitation, the cells were cultured in 25 cm^2^ culture flask. Under the microscope, the growth of the cells was observed. When the cell wall grew more than 60%, the cell passage was carried out. In the ultra-clean table, the old culture medium of cells was sucked out. 2 ml of high temperature sterilized PBS was added and the cells were washed (repeated 3 times). 0.5 ml trypsin was absorbed and added into the culture bottle. The culture bottle was slowly rotated to ensure that the trypsin completely covered the cells. It was observed under a microscope for 1 min at 37 °C. After the cells became transparent and round, 2 ml of the whole medium was added to stop digestion. The wall of the culture bottle was repeatedly gently washed to ensure that the adherent cells were completely mixed into the culture medium. The medium was sucked into a 15 ml centrifuge tube and centrifuged at 1000 rpm for 5 min. In the ultra-clean table, it was necessary to open the centrifuge tube, slowly suck the supernatant into the new 2 ml complete medium, and mix well. Then, 5 ml of complete medium was added into the new culture bottle, 200 µl cell suspension was added into it. The culture flask was placed flat, slowly shaken, and cultured in the incubator with 5% CO_2_ at 37 °C.

20 µl of the suspension was added to the EP tube (Tiangen biochemical technology (Beijing) Co., Ltd., China). After being diluted with PBS for three times, the cells were counted on the cell counting plate (Tiangen biochemical technology (Beijing) Co., Ltd., China) to calculate the cell concentration of the suspension. The cells were seeded into a 6-well plate according to the amount of 10^5^ per well. Each well contained 1.5 ml of complete medium. The plates were slowly shaken and cultured in the incubator at 37 °C and 5% CO_2_.

When the cells grew to more than 60%, the culture medium was replaced by the basic medium without serum and starved for 24 h. The cells were divided into no treatment group (McCoy, 5A basic medium), sodium butyrate pretreatment group (basic medium dissolved sodium butyrate to the final concentration of 0.05 mol/l) and IL-33 (R&D systems, USA) stimulation group (basic medium diluted IL-33 protein to 10^−3^ug/ml, and added into the pore to stimulate HT-29 cells to react for 2 h). One hour before the cells were stimulated with IL-33, sodium butyrate solution was added for pretreatment. One hour later, IL-33 stimulation was performed. After the intervention, the total protein was extracted and the content of IRAK1 protein was detected by Western blotting.

### 2.6. Western blotting detection

(1) Cell total protein extraction: the cell culture medium was aspirated and PBS was used to wash three times. The prepared cell lysate was added. The pipette was used to transfer the pyrolysis liquid to the clean EP pipe. After 30 minutes, the protein was fully cleaved and centrifuged at 12 000 rpm at 4°C for 12 min. After the supernatant was gently absorbed, the protein concentration was measured and the cells were frozen at −80 °C.

(2) BCA method for determining the total protein concentration: first, 1.2 ml protein standard solution was added into 30 mg BSA (bovine serum albumin, Acmec company, China), and then the protein standard solution of 25 mg/ml was prepared. Then, the appropriate amount of protein standard solution was taken and diluted to 1 mg/ml. Then, the BCA working solution was prepared according to the volume relationship of BCA reagent A/BCA reagent B = 50:1, and it was mixed evenly. Then, the standard solution was added into 96 well plates with 0, 1, 2, 4, 8, 12, 16, 20 μl in turn. Then, the standard diluent was added into the hole less than 20 µl, so that the volume of liquid in the hole reached 20 µl. Finally, 200 μl BCA solution was added into each pore, and the reaction was carried out at 37 °C for 25 minutes. The optical density under the condition of 560 nm nanometer wavelength was measured, and the standard curve was drawn to calculate the protein concentration.

(3) Sodium dodecyl sulfate-Polyacrylamide gel electrophoresis (SDS-PAGE): first, 10 ml 10% separation gel and 4 ml 5% concentrated gel were prepared (The reagents purchased from Tiangen biochemical technology (Beijing) Co., Ltd., China). After the glue solidified, it was necessary to clamp the glass plate and the plastic replacement plate in the rack of the charged electrode, put it into the electrophoresis tank, and pull out the comb. After the expression of the protein supernatant was taken out of 30 µl, 10 μl of 5 × sample buffer was added and mixed evenly, and boiled at 100 °C for 10 min. Finally, 40 µl samples were loaded into each well of the gel. Under the voltage of 80 V, bromophenol blue formed a straight line in the glue, and then the voltage was changed to 120 V. When the bromophenol blue ran to the lower edge, the power supply was cut off, and then the membrane was transferred. After the completion of the transfer film, the molecular weight and net optical density of the target band were analyzed by a gel image processing system (Unverbindlicher Verkaufspreis, Germany). The relative expression amount of target protein = gray value OD of target band/gray value of internal reference OD.

### 2.7. Statistical methods

SPSS26.0 software was used to analyze the data, which was expressed by $\overset{ˉ}{\chi }\pm s$ Mann–Whitney U test was used to compare the immunohistochemical scores between groups. Spearman correlation analysis was used to analyze the correlation between immunohistochemistry score and abdominal pain score, and the correlation between IRAK1 content of colon and visceral pain threshold. Kruskal–Wallis H test was used to analyze visceral pain threshold and semi quantitative comparison. The positive rate of immunohistochemistry was analyzed by χ^2^ test.

## 3. Results

It was assumed that sodium butyrate had a regulatory effect on irritable bowel syndrome. The purpose was to analyze the effect of sodium butyrate on IRAK1 at the cellular level and explore the effect of sodium butyrate on IRAK1 protein expression and visceral sensitivity in the colon of irritable bowel syndrome. The following work had been done. First, the IBS mouse model was established, and sodium butyrate enema was used to explore whether sodium butyrate could regulate IRAK1. Then, the positive rate of cells was observed by immunohistochemical staining. Moreover, Western blotting was used to detect the relative protein expression. Finally, the cell experiment was designed. Human colon cancer cell line HT-29 cells was selected and stimulated by IL-33 to up regulate IRAK1 protein expression. Whether the resistance of HT-29 cells to inflammatory factors changed after sodium butyrate pretreatment was analyzed.

### 3.1. Determination of visceral sensitivity in mice by colorectal distention test

**Figure 1.**
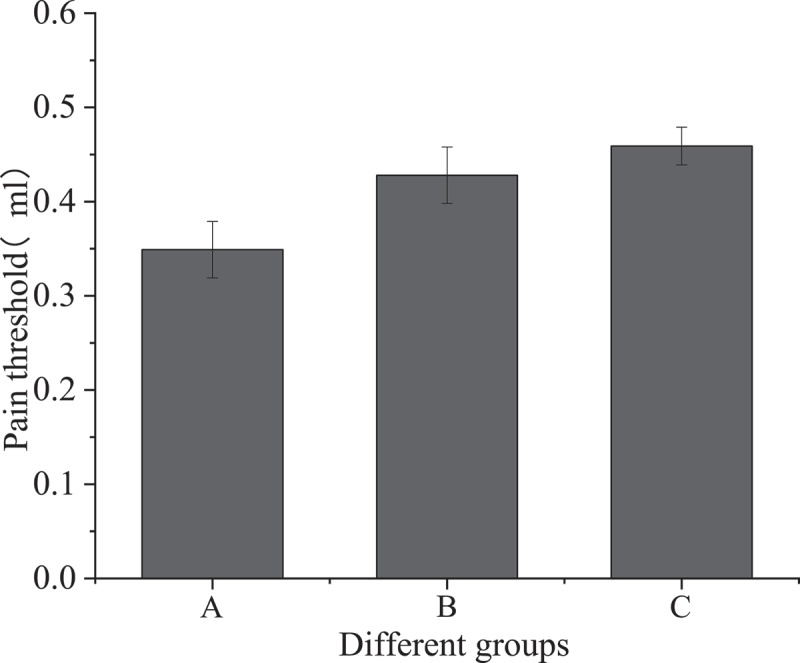
Visceral sensitivity of three groups of mice

Colorectal distention test was performed on three groups of mice to measure visceral pain threshold. The changes in visceral sensitivity were observed as shown in Figure 1.

Figure 1 shows that the visceral pain threshold of the IBS model group was 0.349 ± 0.03, that of the sodium butyrate treatment group was 0.428 ± 0.03, and that of the blank control group was 0.459 ± 0.02. Compared with the IBS model group, the visceral pain threshold of the sodium butyrate treatment group was increased, and the difference was statistically significant (P < 0.05); compared with the blank control group, the visceral pain threshold of the IBS model group was significantly different (P < 0.001); compared with the blank control group, the pain threshold of the sodium butyrate treatment group was not statistically significant (P > 0.05).

### 3.2. Expression of IRAK1 and p-IRAK1 protein in intestinal epithelial cells of mice in each group

**Figure 2.**
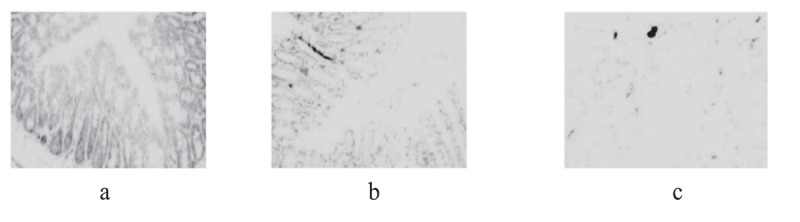
Staining results of colon transverse section of three groups of mice (a): IBS model group; (b): sodium butyrate group; (c): blank control group)

Immunohistochemical staining method was used, that is, the specific antibody was used to stain the transverse section of the colon of three groups of mice, and the expression of IRAK1 protein in colonic epithelial cells of mice in each group was observed, as shown in Figure 2(a–c).

In Figure 2, IRAK1 protein-specific staining of intestinal epithelial cells in the IBS model group was compared with the sodium butyrate treatment group and the blank control group, the staining was deeper, and the proportion of positive cells was increased. There was no significant difference in specific protein staining between the sodium butyrate treatment group and the blank control group.

**Figure 3.**
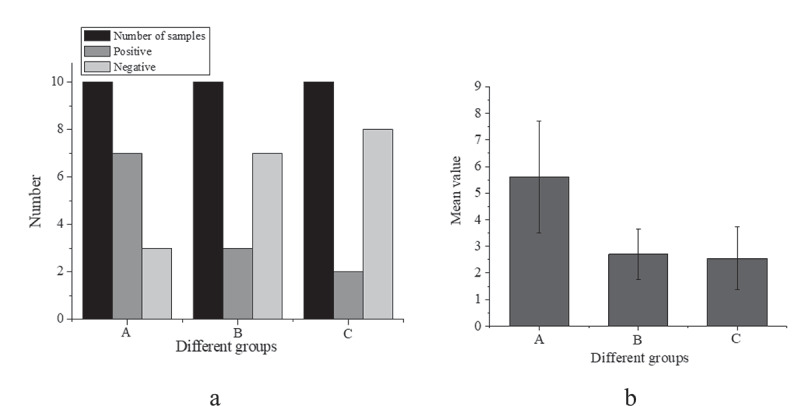
Expression of IRAK1 protein in colon epithelial cells of mice ((a): comparison of sample number, positive and negative number of mice in three groups; (b): comparison of mean value of three groups of mice)

The expression of IRAK1 protein in colonic epithelial cells of the mice in each group was evaluated by the pathological scoring method, as shown in Figure 3(a and b).

Figure 3 suggests that the number of positive cells in the IBS model group was 7, that in the sodium butyrate group is 3, and that in the control group is 2; the trend of the number of negative cells and positive cells is opposite, and the number of negative cells in A, B, and C three groups is 3, 7 and 8 in turn. This indicates that the expression of IRAK1 protein in colonic epithelial cells of three groups was different, and the difference was statistically significant (H = 8.799, P < 0.001). Compared with the control group (2.55 ± 1.19), the expression of IRAK1 protein in colon epithelial cells of IBS model mice (5.59 ± 2.11) was increased, and the difference was statistically significant (P < 0.001). Compared with the IBS model group, the expression of IRAK1 protein in the sodium butyrate treatment group (2.69 ± 1.19) was significantly decreased, and the difference was statistically significant (P < 0.05). There was no significant difference between the sodium butyrate treatment group and the blank control group (P > 0.05).

### 3.3. Correlation between IRAK1 protein content in colon tissue and visceral sensitivity

**Figure 4.**
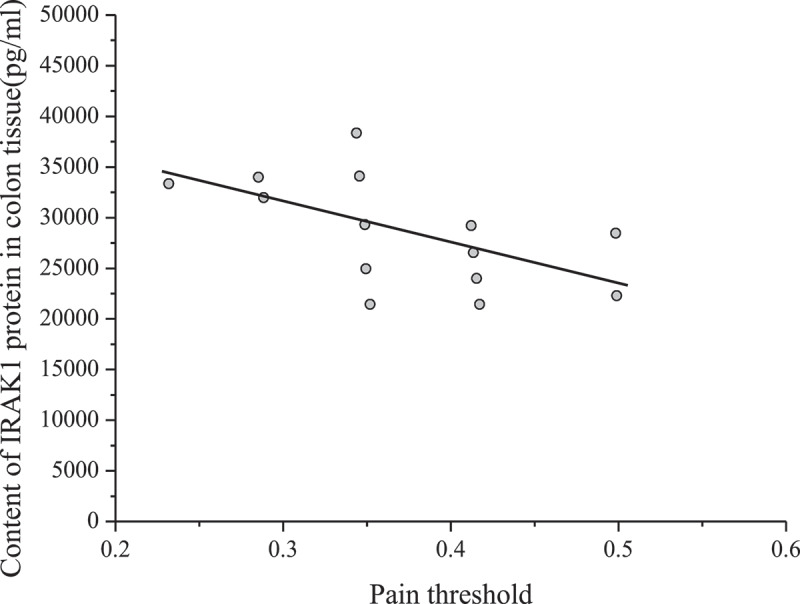
Relationship between IRAK1 content in colon tissue and visceral pain threshold in mice

Spearman rank correlation was used to analyze the relationship between the content of IRAK1 in colon tissue and visceral pain threshold. The results are shown in Figure 4.

Figure 4 shows that the content of IRAK1 protein in colon tissue of mice was inversely proportional to the pain threshold of the colorectal distention test (correlation coefficient r = – 0.653, P < 0.001). The pain threshold was inversely proportional to visceral sensitivity. Therefore, the content of IRAK1 protein in colon tissue of experimental mice was in direct proportion to visceral sensitivity.

### 3.4. Effect of sodium butyrate on IRAK1 protein expression in HT-29 cells

**Figure 5.**
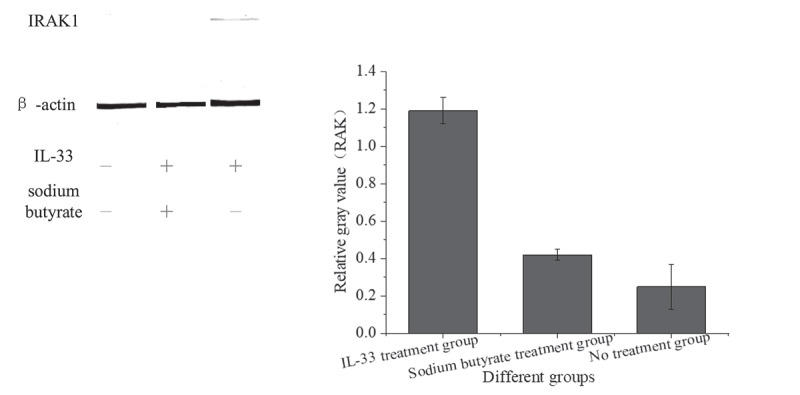
IRAK1 protein expression in HT-29 cells of different groups

Western blotting was used to detect the expression of IRAK1 protein in HT-29 cells after intervention with sodium butyrate and IL-33, as shown in Figure 5 below.

Figure 5 shows that the expression of IRAK1 protein in HT-29 cells stimulated by IL-33 was higher than that in the no treatment group (P < 0.001). The cells pretreated with sodium butyrate were stimulated with the same IL-33, and the expression of IRAK1 protein was decreased (P < 0.001).

## 4. Discussion

Butyric acid can inhibit the diseases caused by intestinal inflammation, which is an important material to repair intestinal mucosa and inhibit the spread of inflammation. In inflammatory bowel disease (IBD), relevant experiments showed that sodium butyrate enema could alleviate the intestinal inflammation of IBD [11]. IBS animal model was established. A small dose of sodium butyrate enema was used, and the butyric acid in the intestinal tract of mice was kept close to the normal value. The results of visceral sensitivity-related experiments showed that sodium butyrate intervention could improve visceral hypersensitivity of IBS mice, and the expression of IRAK1protein in colon tissue of mice was reduced, which was contrary to the research results of Russo et al. (2016) [12]. High dose sodium butyrate enema was conducted in mice for 7 consecutive days, which made mice hyperalgesia and produces non-inflammation-related visceral hypersensitivity. In order to further prove the relationship between IRAK1protein and visceral sensitivity, a colorectal distention test was conducted to measure the relationship between visceral pain threshold and IRAK1 protein expression. The results showed that the content of IRAK1protein in colon tissue of mice was inversely proportional to visceral pain threshold, that was, the higher visceral sensitivity of mice, the more IRAK1 content in the colon, which was consistent with the research results of Yup et al. (2016) [13]. Compared with the non-stress group, the number of fecal particles in the stress group was significantly increased, and the visceral sensitivity was higher.

IL-33 could bind to the ST2 receptor, which could recruit IRAK1 protein, phosphorylate IL-33 and activate the downstream signaling pathway. In order to explore the relationship between sodium butyrate and IRAK1 protein, cell experiments were carried out. The colon epithelial cell line HT-29 was stimulated with an appropriate amount of IL-33. Western blotting was used to detect the expression of IRAK1 protein. The results showed that IL-33 stimulated HT-29 cells to increase IRAK1 protein expression, which was consistent with the research results of Gautier et al. (2016) [14]. IL-33 cytokines induced endothelial cells to express and inhibited inflammatory response related proteins. The cells that were about to be stimulated by IL-33 were pretreated with sodium butyrate. The results showed that sodium butyrate pretreatment could reduce the increase of IRAK1 protein. Sodium butyrate could alleviate the inflammatory response of colonic epithelial cells stimulated by inflammatory factors.

## 5. Conclusion

The expression of IRAK1 protein in the intestinal tract of IBS mice can increase visceral hypersensitivity. Sodium butyrate intervention can reduce the expression of IRAK1 protein and relieve visceral hypersensitivity of IBS. Under the stimulation of IL-33 inflammatory factors, sodium butyrate pretreatment can reduce the increase of IRAK1 protein in HT-29 cells. There are still some deficiencies, like the limited sample size, which will make the research results not convincing enough. This is where the follow-up research needs to be improved.

## Supplementary Material

Supplemental Material

## Data Availability

Some or all data, models, or code generated or used during the study are available from the corresponding author by request.

## Highlights

- IL-33 stimulates HT-29 cells to promote the increase of IRAK1 protein expression.
- Sodium butyrate can improve visceral hypersensitivity of IBS mice by reducing IRAK1 protein expression in colon.
- The higher the visceral sensitivity of mice is, the more IRAK1 content in colon is.
- Sodium butyrate is involved in alleviating the inflammatory response of colonic epithelial cells.
