# Supplementary figures and images for: Effect of sodium butyrate regulating IRAK1 (interleukin-1 receptor-associated kinase 1) on visceral hypersensitivity in irritable bowel syndrome and its mechanism
Source: Bioengineered. 2021 Apr 27;12(1):1436–44. doi: 10.1080/21655979.2021.1920324 (PMC8806247; doi:10.1080/21655979.2021.1920324)

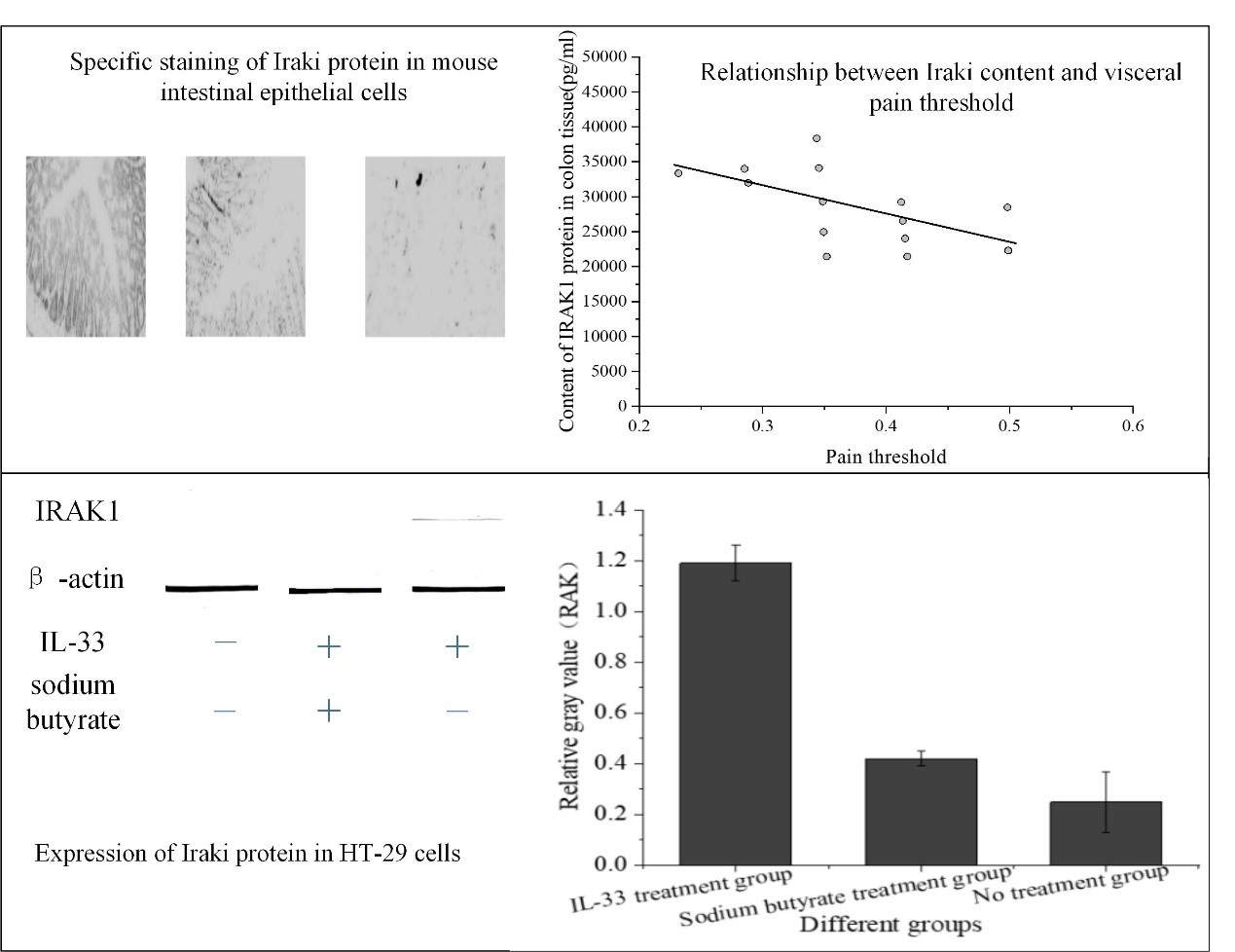

Supplement: Supplemental Material [file KBIE_A_1920324_SM4033.jpg]
